# Winter dynamics of functional diversity and redundancy of riffle and pool macroinvertebrates after defoliation in a temperate forest stream

**DOI:** 10.3389/fmicb.2023.1105323

**Published:** 2023-03-06

**Authors:** Lu Wang, Lixian Xia, Jiaxu Li, Linglin Wan, Haijun Yang

**Affiliations:** ^1^School of Ecology and Environmental Science, Yunnan University, Kunming, China; ^2^Department of Ecology, Institute of Hydrobiology, Jinan University, Guangzhou, China

**Keywords:** litter patch, freezing and thawing, functional trait, functional redundancy, hydrological disturbance

## Abstract

Headwater streams are highly heterogenous and characterized by a sequence of riffles and pools, which are identified as distinct habitats. That higher species richness and density in riffles than in pools is considered a general pattern for macroinvertebrates. As temperate winters can last long up to half a year, however, macroinvertebrate communities of riffles and pools may assemble differently under ices or snows. Particularly, defoliation concentrating in autumn can largely change habitats in both riffles and pools by litter patching. According to the absence or presence of litter patches, there exist four types of subhabitats, i.e., riffle stones, riffle litters, and pool sediments, pool litters, which are selectively colonized by macroinvertebrates. To study the spatial pattern and temporal dynamics of colonization, macroinvertebrates were surveyed in a warmer temperate forest headwater stream in Northeast China during four periods: autumn, pre-freezing, freezing, and thawing periods. Our study focused on functional trait composition, functional diversity and functional redundancy of macroinvertebrate communities. The colonization of macroinvertebrates was found to be significantly different in these subhabitats. Riffle stones supported higher taxonomic and functional diversities than pool sediments; litter patches supported higher total macroinvertebrate abundance and higher functional redundancy than riffle stones or pool sediments. The functional trait composition changed significantly with seasonal freeze-thaw in both riffle stones and pool sediments, but not in litter patches. Macroinvertebrate community in litter patches showed seasonal stability in taxonomic and functional diversities and functional redundancy. Thus, this study strongly highlights that litter patches play an important role structuring macroinvertebrate community over winter, supporting high abundance and maintaining functional stability.

## 1. Introduction

Headwater streams are ubiquitous in river landscapes and are important sources of biota for downstream reaches, and critical sites for maintaining the ecological integrity and health of whole river networks (Clarke et al., 2008; Finn et al., 2011; Callisto et al., 2021). Understanding their biological diversity and community assembly is fundamental to monitoring and management (Clarke et al., 2008; Finn et al., 2011). Headwater streams are highly heterogenous and characterized by a sequence of riffles and pools. Riffles and pools are identified as distinct habitats, and significantly different in physical features including flow, depth, slope, and substrate composition (Gordon et al., 1992; Merritt and Cummins, 1996; MacWilliams et al., 2006). Studies of riffles and pools have shown a general pattern for macroinvertebrate communities: species richness and density are higher in riffles than in pools (Scullion et al., 1982; Logan and Brooker, 1983; Brown and Brussock, 1991). Additionally, macroinvertebrate composition of functional feeding groups is different between the two habitat types, for example, more scrapers in riffles and more gather-collectors in pools (Cummins, 2016). However, such a pattern changes largely with latitude, season, flow regime, and environmental stress (Boulton and Lake, 1992; Carter and Fend, 2001; Bogan and Lytle, 2007; Mendes et al., 2017; Herbst et al., 2018). For example, discharge regime has been found to affect largely macroinvertebrate community composition in snowmelt-dominated streams (Carter and Fend, 2001; Herbst et al., 2018).

Although there have been many studies from temperate streams, how biological communities of riffles and pools assemble under surface ices in temperate winter remain unclear. In high latitudes, winter is rather long and even lasts up to ≥half a year. However, most seasonal studies usually have a low time-resolution for such a long winter. In temperate streams, particularly, leaves from riparian vegetation intensively fall in autumn and are accumulated in the riffles and pools (Kobayashi and Kagaya, 2002, 2004). As leaves are not only an important food source, but also can modify habitat heterogenicity, sudden accumulated leaves and thereafter decomposition can influence the structure and dynamics of macroinvertebrate community during winter (Richardson, 1992; Mendes et al., 2017; Al-Zankana et al., 2021). Leaves are unevenly distributed in riverbeds and form so-called litter patches in both riffles and pools. In riffles, litter patches easily occur at the upstream face of flow obstacles (such as stones, and branches) and usually have higher leaf mass. In pools, litter patches occur in places with low water currents and have higher mass of wood and small litter fragments. According to litter’s presence or absence, there are four types of distinct subhabitats: riffle stones, riffle litters, and pool sediments, pool litters. The four subhabitats have distinct physical and chemical conditions, and can be colonized selectively by macroinvertebrates. Such colonization significantly depends on rainfall and discharge (Buss et al., 2004).

Within a stream, difference in macroinvertebrate communities between riffles and pools is dependent of environmental conditions such as flow regimes and food supply (Scullion et al., 1982; Brown and Brussock, 1991). Several studies reported that difference in species richness is not always significant, but the difference in quantitative composition is more common (Mendes et al., 2017). During a long temperate winter that can be divided into pre-freezing, freezing, and thawing periods, the difference in macroinvertebrates between riffles and pools is expected to change largely from the autumn just after defoliation toward water freezing and snow thawing next spring.

For the assembly of macroinvertebrate communities, both classical river continuous concept and habitat templet theory suggest that species sorting or environmental selection be stronger over smaller spatial extents (Vannote et al., 1980; Townsend and Hildrew, 1994; Hamilton et al., 2020). As a local filter, environmental selection retains species with suitable functional traits, which are morphological, biochemical, physiological, structural, phenological, or behavioral features that influence performance or fitness (Nock et al., 2016). Functional diversity, a component of biodiversity, is defined as the functional trait differences between organisms present in a community, mostly including functional richness, evenness, and divergence (Mason et al., 2005, 2013). The functional diversity of a local community indicates species heterogeneity and is strongly associated with its performance in environmental change (Mason et al., 2005, 2013). Thus, trait–based assessment (i.e., functional trait composition analysis) is likely to detect sensitively the structural and functional difference between riffles and pools (Herbst et al., 2018). At community level, functional redundancy is defined as the fraction of taxonomic diversity not expressed by functional diversity (Ricotta et al., 2016). It provides an important measure indicating community assembly in viewpoint of functional traits (Ricotta et al., 2020). Although litter patches are attractive to many groups of macroinvertebrates, they tend to decrease substrate heterogeneity in both riffles and pools, and may reduce functional diversity and increase functional redundancy.

For temperate deciduous broad-leaved forests, the defoliation concentrates in autumn, macroinvertebrates immediately colonize into the litter patches, and specially, shredders intensively involve and facilitate litter decomposition (Cummins et al., 1989; Kobayashi and Kagaya, 2005, 2009). Usually, it takes months for litters to be completely decomposed (Gessner et al., 1999). Such decomposition occurring hiddenly under ices or snows may be limited by rather low water temperature. Macroinvertebrate richness and density increase with litter palatability and may peak in the middle and even late winter. Toward next spring, snowmelt can result in increasing discharge that can change habitat stability, promoting passive or active dispersal of many species. Species colonized into the litter substrates of both riffles and pools become biota sources for downstream. Therefore, macroinvertebrate assemblages experience a highly dynamic succession (Wang, 2020).

**FIGURE 1:**
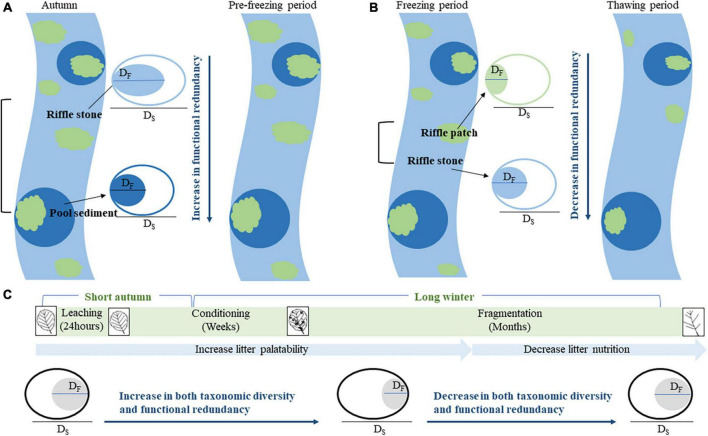
A hypothesized variation of functional diversity and redundancy between four subhabitats (riffle stone, pool sediment, riffle litter, and pool litter) and between four periods (autumn, pre-freezing, freezing, and thawing periods). **(A)** The difference in functional redundancy between riffle stones and pool sediments; **(B)** the difference in functional redundancy between riffle sediments and riffle litters; **(C)** the temporal variation of functional redundancy from autumn to next spring. D_S_, taxonomic diversity, indicated by an open ellipse; D_F_, functional diversity, shown by a filled ellipse, D_F_ ≤ D_S_. Functional redundancy = 1–D_F_/D_S_.

In this study, we aim to test three hypotheses that highlight difference in functional diversity and redundancy of macroinvertebrate communities between riffles and pools (Figure 1). (1) Riffles have higher habitat heterogeneity than pools, thus, host richer macroinvertebrate species and higher functional diversity. Since environmental selection has greater impact on functional traits than species themselves, higher functional diversity leads to lower functional redundancy in riffles than in pools. (2) For both riffles and pools, litter patches provide macroinvertebrates with more foods but lower substrate heterogeneity. The reduced environmental selection and limited competition under low temperature will result in lower functional diversity and higher functional redundancy. (3) From the pre-freezing to freezing periods, food that increases with litter decomposition supports high richness and density of colonized macroinvertebrates. During this period, both low temperature and high food resource reduce interspecific competition, and tends to decrease functional diversity and increase functional redundancy. From the freezing to thawing periods, however, leaf nutrition decreased with litter decomposition, the richness and density of colonized macroinvertebrates decrease, and nutritional limitation promoted interspecific competition, resulting in increase in functional diversity and decrease in functional redundancy.

In the present study, we test the three hypotheses by examining macroinvertebrates in a warmer temperate stream of the Songhua River, Jilin Province, Northeast China. We limited this study in a single stream so that all local communities surely share a common species pool and all sites have a common regional background, especially the same riparian vegetation. The resulted dataset can reduce the complex influence of multiple factors. This study provides a case study of the dynamics of stream macroinvertebrate assembly associated with defoliation in warmer temperate.

## 2. Materials and methods

### 2.1. Study area

**FIGURE 2:**
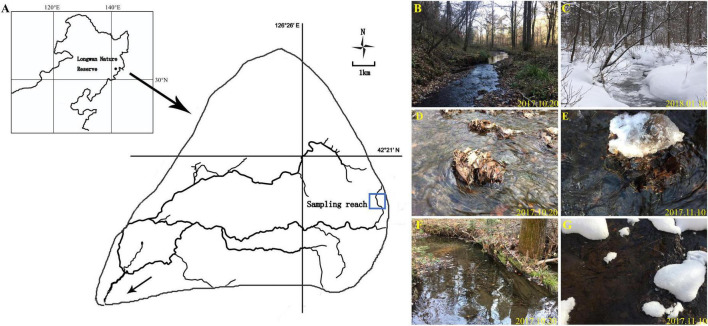
Location of sampling reach **(A)**; sampling reach with sequence of riffles and pools in the autumn **(B)**; sampling reach in the winter **(C)**; riffle litter in the autumn **(D)**; riffle litter in the winter **(E)**; pool litter in the autumn **(F)**; pool litter in the winter **(G)**.

The filed investigation was conducted in a forest headwater stream of the Songhua River Basin, of Northeast China, which is located in the Longwan Nature Reserve (126^°^13′55″–126°13′55″N, 42^°^16′20″–42^°^26′57″E). The river basin has a warm temperate and continental monsoonal climate, with a mean annual precipitation of about 700 mm. The mean annual water temperature is about 7^°^C, and the monthly water temperature varies from 0.5^°^C in January in winter to 15^°^C in July in summer. Winter here lasts for 5 months from November to April each year, during which approximately 70% of the surface stream water is frozen. The riparian vegetation is dominated with tree species: *Acer mono*, *Tilia amurensis*, *Quecus mongolica*, *Ulmus pumila*, and *Populus davidiana*. In this study, a 500 m reach was selected to consist primarily of fast-flowing riffles and slow-flowing pools (Figure 2A). From autumn to next early spring, the riverbeds are covered with leaves or litters that form litter patches in both riffles and pools. Following the classification by Buss et al. (2004), we defined four common substrates: riffle stones and pool sediments, riffle litters, and pool litters (Figures 2B–G).

### 2.2. Sampling and identification of macroinvertebrates

Macroinvertebrates and litter patches were investigated across four periods: autumn (mid October of 2017), pre-freezing (early November of 2017), freezing (early January of 2018), and thawing periods (early March of 2018). The sampling was performed in a 500 m reach that consists primarily of fast-flowing riffles and slow-flowing pools. In each period, four litter patches from four pools and four litter patches from four riffles were sampled. For riffle litters, the components of litter patches and macroinvertebrates were collected with a Surber sampler (30 × 30 cm, 500 μm mesh size) because patches size (area covered by litter) was less than 900 cm^2^. Organic matter inside the sampler except for large branches was washed into the net. The size of sampled litter patches was recorded. For litters and macroinvertebrates in pool patches, a square-cut cushion of sponge rubber (50 × 50 cm with an inner 20 × 20 cm opening) was putted on litters for quantitative sampling. Organic matter inside the opening was washed into the D-frame net (500 μm mesh size). In each period, six riffles and six pools were randomly selected for collecting macroinvertebrates of riffle stones and pool sediments. Habitats for riffle stones and pool sediments were identified visually based on velocity, depth, particle size, and moss cover. One sample of macroinvertebrates was collected and integrated for each riffle or pool at three microhabitats with a Surber net (30 × 30 cm, 500 μm mesh size). All the macroinvertebrates were stored in 75% ethanol.

In the laboratory, all macroinvertebrates were individually picked from the detritus and other materials from each sample. All litter samples were washed through nested sieves (16 and 1 mm), and the contents of these sieves were separated into litter and macroinvertebrates. Macroinvertebrates were identified to the genus level, except for Chironomidae, which was taxonomically rich and identified as a subfamily. Identification and counting of taxa were performed by a stereoscopic microscope using monographs, publications, and other relevant literature (Morse et al., 1994; Wiggins, 1996; Thorp and Covich, 2001). All litters were classified into three categories: coarse particulate organic matter (CPOM: >16 mm), leaves (16 mm), and small woody detritus (SWD: 16–100 mm). The litters in each category were dried at 60^°^C for 48 h and weighed.

### 2.3. Measurements of environmental variables and litter patches characteristics

Environmental variables were synchronously measured at riffles and pools. The water velocity (Vel) was measured using a portable velocity analyzer. Water depth was estimated using a graduated stick. Water temperature (Temp), pH, turbidity (Turb), conductivity (Cond), and dissolved oxygen (DO) were measured using a portable water quality analyser (YSI). Substrates were quantified by visually estimating the percentage of boulders, cobbles, pebbles, gravel and sand following the protocol established by Cummins (1962). In each period, six riffle patches and six pool patches were randomly selected for measuring. Detritus area (cm^2^), detritus height (cm), and water depth (cm) were recorded, and current velocity (m/s) just above the patches was measured using a portable current meter.

### 2.4. Measures of taxonomic diversity, functional diversity, and functional redundancy

Taxonomic diversity was measured with taxon richness and Simpson’s diversity. Functional diversity was measured using Rao’s diversity. To calculate functional diversity, 21 traits in six types (Supplementary Table 1) were chosen from the database information published by Poff et al. (2006), Tomanova and Usseglio-Polatera (2007). The chosen traits represent the dimensions of the ecological niche of macroinvertebrates, including life history (voltinism), mobility (swimming ability), morphology (shape), and ecology (rheophily, habitat, trophic habits), and have been proved to be sensitive to the environmental conditions, such as physical environment condition and food resource (Cummins, 1974; Tomanova and Usseglio-Polatera, 2007). Functional trait dissimilarity between taxa was quantified using the gawdis distance with the “gawdis” function in the R package (de Bello et al., 2021). Simpson’s diversity (D), Rao’s diversity (Q), and the taxon-level vulnerability with the “uniqueness” function were calculated in the ade package (Ricotta et al., 2016). Following a framework proposed by Ricotta et al. (2016, 2021), Pavoine and Ricotta (2019), functional redundancy was measured as the fraction of taxonomic diversity not expressed by functional diversity. Finally, we calculated functional redundancy (FR): FR = (D–Q)/D (Ricotta et al., 2016). The methodology of calculating functional diversity and functional redundancy was described in detail in Wang et al. (2023). All diversity analyses were performed using R v4.2.0.

### 2.5. Statistical analysis

Two-way analysis of variance (ANOVA) was applied to determine the differences in environmental variables between riffles and pools and among the four periods. The difference in the composition of macroinvertebrate communities was tested among the and four periods between riffle litters and pool litters, between riffle stones and riffle litters, and between pool sediments and pool litters by two-way analysis of similarities (ANOSIM) based on the Bray-Curtis dissimilarity matrix, separately. Then, SIMPER (similarity percentages-species contributions) was performed to determine the species that most contribute to the differences. The difference in relative abundance of each functional trait, species richness, density, Simpson’s, Rao’s diversity and functional redundancy among four periods and between riffle litters and pool litters, between riffle stones and riffle litters, and between pool sediments and pool litters were also detected by two-way ANOVA. Where significant ANOVA result was obtained (*p* < 0.05), Tukey’s multiple comparisons tests were conducted. Nested ANOVA analysis was applied to determine the difference in species richness, Simpson’s, Rao’s diversity and functional redundancy between riffles (include riffle stones and riffle litters) and pools (include pool sediments and pool litters) in each period. The ANOVA and Tukey’s test were performed using SPSS software (version 21.0). The two-way similarity (ANOSIM) and similarity percentages-species contributions were conducted using PAST software (version 3.0) (Hammer et al., 2001).

Redundancy analysis (RDA) was run to check the variables that influence community variation of the macroinvertebrate communities. Environmental variables included velocity, depth, substrate composition (boulders, cobbles, pebbles, gravel, and sand%), total litter abundance and four litter components (CPOM abundance, Leaves abundance, and SWD abundance). Species’ population density was Hellinger transformed and environmental variables were standardized prior to RDA. The significance of the full model of RDA was test with the ANOVA function. A forward selection procedure was conducted with the ordiR2step function to select the significant variables. Redundancy analysis was run in the vegan package (Legendre and Legendre, 2012). The hierarchical partitioning method was used to distinguish a single variable’s contribution *via* the rdacca.hp package (Lai et al., 2022).

## 3. Results

### 3.1. Environment conditions

**FIGURE 3:**
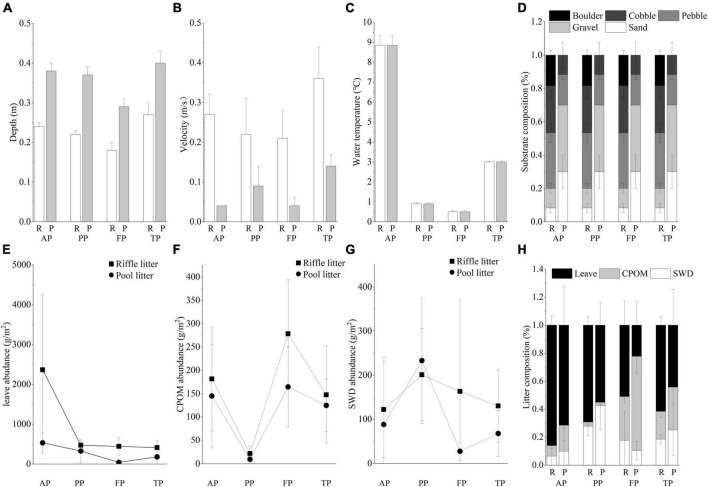
Physical environmental conditions: **(A)** depth, **(B)** velocity, **(C)** water temperature, **(D)** substrate composition in riffles and pools during the four periods; and litter composition: **(E)** leave abundance, **(F)** CPOM abundance, **(G)** SWD abundance, and **(H)** relative abundance of each litter type in the riffle and pool litter patches during the winter. R, riffles; P, pools; AP, the autumn period; PP, the pre-freezing period; FP, the freezing period; TP, the thawing period; CPOM, coarse particulate organic; SWD, small woody detritus.

Physical environmental variables exhibited significant differences between riffles and pools (Figures 3A–D). Water flow velocity was higher in riffles, while water depth was higher in pools. Substrates in riffles were mainly composed of boulders and cobble, but those in pools had higher proportion of sand and gravel. The characteristics and composition of litter patches were significantly different between riffles and pools. Pool litters had higher litter area (Figures 3E–H and Supplementary Table 2), but lower litter abundance than riffle litters. Pool litters had higher relative abundance of coarse particulate organic matter (CPOM), while riffle litters had higher relative abundance of leave.

Toward the winter, water temperature decreased extremely, and the stream below the ice surface was characterized by low water depth and low water velocity. Subsequently, in the thawing period, the increased temperature accelerated ice-snow melting, resulting in significant increase in water velocity. However, there was no obvious change in substrate composition during the whole winter. Regarding litter composition, the relative abundance of leaves was highest in the autumn, then decreased gradually, while the relative abundance of CPOM was highest in the freezing period.

### 3.2. Macroinvertebrate composition

In total, 50 taxa from 9 orders and 31 families were identified in the sampled macroinvertebrates (Supplementary Table 3): 36 taxa occurred in riffle stones, 29 in pool sediments, 33 in riffle litters, and 32 in pool litters. *Ephemerella*, *Taenionema*, *Hydropsyche*, Orthocladiinae, Chironominae, and *Gammarus* dominated in riffle stones, while *Ephemerella*, Orthocladiinae, Chironominae, and *Pseudamophilus* did in pool sediments. *Ephemerella*, *Utaperla*, *Taenionema*, Tanypodinae, Orthocladiinae, and *Gammarus* were dominant groups in riffle litters, while *Utaperla*, *Taenionema*, Tanypodinae, Orthocladiinae, and Chironominae were dominant in pool litters.

**FIGURE 4:**
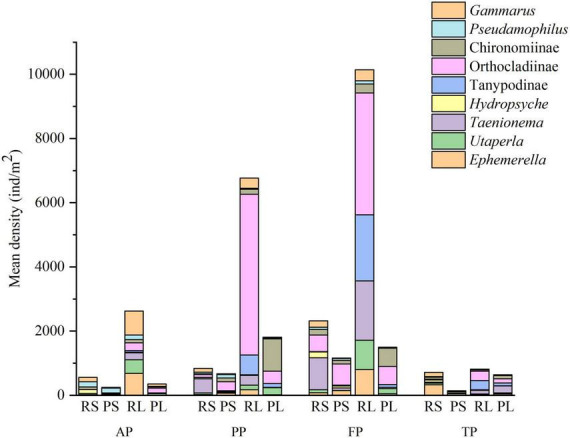
Change in the mean density of main groups that caused spatial and temporal differences of community composition *via* similarity percentages (SIMPER) analysis. RS, riffle stones; PS, pool sediments; RL, riffle litters; PL, pool litters; AP, autumn period; PP, pre-freezing period; FP, freezing period; TP, thawing period.

ANOSIM analysis showed considerable difference in macroinvertebrate communities between riffle stones and pool sediments in each period (*p* < 0.05), between riffle stones and riffle litters in each season (*p* < 0.05), and between pool sediments and pool litters during the autumn and pre-freezing periods (*p* < 0.05). Both riffle stone and pool sediment communities showed significant temporal variation between different periods (*p* < 0.05). Riffle litter community significantly changed from the autumn to the pre-freezing period and from the freezing to the thawing periods (*p* < 0.05). However, pool litter community changed significantly only from the autumn to the pre-freezing periods (*p* < 0.05). SIMPER analysis showed the density variation of Chironominae, *Utaperla*, *Taenionema*, and Tanypodinae most contributed to spatial and temporal differences in community composition (Figure 4).

**FIGURE 5:**
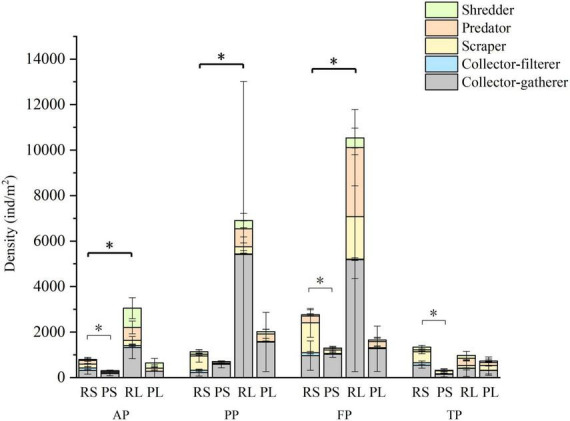
Density of macroinvertebrates and functional feeding groups in the four types of substrates during the four periods. Light black asterisk indicated significant difference between riffle stones and pool sediments; dark black asterisk indicated significant difference between riffle stones and riffle litters. RS, riffle stones; PS, pool sediments; RL, riffle litters; PL, pool litters; AP, the autumn period; PP, the pre-freezing period; FP, the freezing period; TP, the thawing period.

The density of macroinvertebrates, especially collector-filterers, scrapers and shredders were significantly higher in riffle stones than in pool sediments (*p* < 0.05, Figure 5). Riffle litters supported higher macroinvertebrate density than riffle stones. Regarding functional feeding groups, predator density was significant higher in riffle litters than in riffle stones in each period (*p* < 0.05); collector-gatherer and shredder density were higher in riffle litters from the autumn to the freezing periods (*p* < 0.05); however, collector-filterer density was higher in riffle stones than in riffle litters from the pre-freezing to the thawing periods (*p* < 0.05). However, there were no significant difference in density of each functional feeding group between pool sediments and pool litters.

Collector-gatherer density was highest during the freezing period in both riffle stones and pool sediments; scrapers and predator density be highest during the freezing period in riffle stones. In riffle litters, collector-filterer density was highest during the autumn; while predator density was highest in the freezing period. In pool litters, the densities of macroinvertebrates and their individual functional feeding group has no significant difference between the four periods.

RDA showed that the full model (adjR^2^ = 0.120, *p* = 0.001) significantly explained the variation in total community structure. The significant variables, i.e., velocity, Boulders%, and total litter abundance, explained 3.65, 4.99, and 3.38% of the total variation of the communities, respectively (Supplementary Figure 1).

### 3.3. Functional trait composition

**FIGURE 6:**
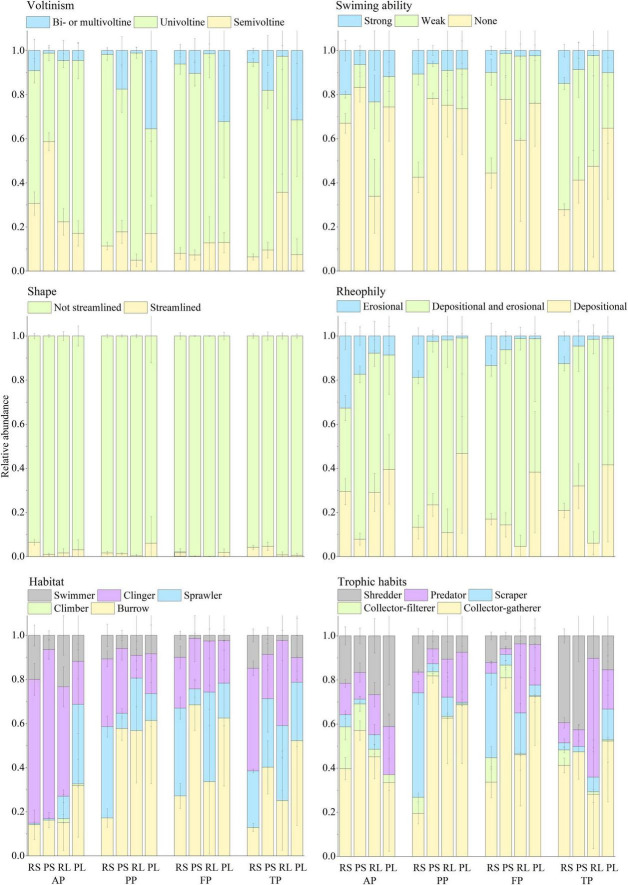
Relative abundance (Mean ± SD) of taxa belonging to different trait states for each trait group in the riffle stones, the pool sediments, the riffle litters, and the pool litters during different period. RS, riffle stones; PS, pool sediments; RL, riffle litters; PL, pool litters; AP, the autumn period; PP, the pre-freezing period; FP, the freezing period; TP, the thawing period.

There was significant difference in functional trait composition between riffle stones and pool sediments, and between riffle stones or pool sediments and their litter patches (Figure 6). Pool sediment community was characterized by higher relative abundance of “Bi- or multivoltine,” “None swim,” “Burrower,” and “Collector-gatherer” taxa; while riffle stone community had more relative abundance of “Erosional” and “Scraper” taxa. Higher relative abundance of “Erosional,” “Collector-filterer,” and “Shredder” taxa occurred in riffle stones than in riffle litters. Higher relative abundance of “Depositional” and “Predator” taxa occurred in pool litters than in pool sediments.

Functional trait composition showed a clear temporal change in both riffle stones and pool sediments. In riffle stones, the relative abundance of “Semivoltine” groups decreased but that of “Univoltine,” “weak swimming,” and “Depositional and Erosional” groups increased significantly from the autumn to the pre-freezing periods; “Shredder” decreased from the freezing to the thawing periods. In pool sediments, the relative abundance of “Erosional” groups decreased but “Burrower” and “Collector-gatherer” groups increased significantly from the autumn to the pre-freezing period; whereas the relative abundance of “Burrower” and “Collector-gatherer” decreased, but “weak swimming,” “Streamlined,” and “Shredder” groups increased from the freezing to the thawing periods. However, functional trait composition in both riffle litters and pool litters did not change significantly over the four periods.

### 3.4. Taxonomic and functional diversity and functional redundancy

**FIGURE 7:**
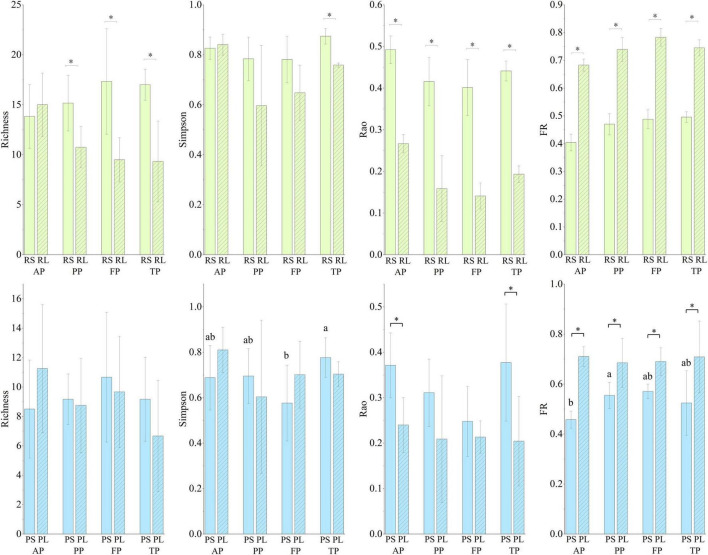
Richness, Simpson’s, Rao’s, and functional redundancy (FR) in the four types of substrates during the four periods. Light black asterisk indicated significant difference between the riffle stones and the riffle litters (*p* < 0.05); dark black asterisk indicated significant difference between the pool sediments and the pool litters (*p* < 0.05); different small letters represent significant difference in Simpson’s diversity and functional redundancy among periods in the pool sediments (*p* < 0.05). RS, riffle stones; PS, pool sediments; RL, riffle litters; PL, pool litters; AP, the autumn period; PP, the pre-freezing period; FP, the freezing period; TP, the thawing period.

Taxonomic and functional diversity and functional redundancy of macroinvertebrate communities showed significant difference between subhabitats (Figure 7 and Supplementary Table 4). Taxonomic richness colonized was significantly higher in riffle stones than in pool sediments, and higher in riffle stones than in riffle litters. Simpson’s diversity was significant higher in riffle stones than in pool sediments during the autumn and the freezing periods. Rao’s diversity was significantly higher in riffle stones than in pool sediments, and significant higher in riffle stones or pool sediments than their litter patches. Functional redundancy did not show significant difference between riffle stones and pool sediments, but was significant higher in litter patches than in both riffle stones and pool sediments.

Simpson’s diversity and functional redundancy showed a significant seasonal variation in pool sediments (Figure 7), where Simpson’s diversity decreased significantly during the freezing period, and functional redundancy increased significantly during the pre-freezing period.

The difference in richness, Simpson’s diversity, Rao’s diversity and functional redundancy between riffles and pools *via* the nested ANOVA analyses showed similar results with the difference between riffle stones and pool sediment *via* the two-way ANOVA (Table 1 and Supplementary Table 4).

**TABLE 1:** Nested analysis of variance (ANOVA) for present-absent litter and habitat (riffle-pool) effects on richness, Simpson, Rao, and functional redundancy (FR) in each period.

Period	Factor	Richness			Simpson			Rao			FR		
		**df**	**F**	***P*** **-value**	**df**	**F**	***P*** **-value**	**df**	**F**	***P*** **-value**	**df**	**F**	***P*** **-value**
Autumn period	Present-absent litter	1	1.06	0.32	1	1.13	0.31	1	52.11	0.00	1	143.78	0.00
Pre-freezing period	Riffle-pool	2	7.71	0.01	2	4.88	0.03	2	9.59	0.00	2	2.16	0.16
	Present-absent litter	1	5.70	0.04	1	1.32	0.27	1	13.85	0.00	1	39.61	0.00
Freezing period	Riffle-pool	2	9.18	0.00	2	0.30	0.74	2	3.19	0.08	2	4.98	0.03
	Present-absent litter	1	6.71	0.03	1	0.05	0.83	1	18.90	0.00	1	84.92	0.00
Thawing period	Riffle-pool	2	4.19	0.05	2	3.45	0.07	2	7.23	0.01	2	3.91	0.06
	Present-absent litter	1	8.76	0.02	1	13.87	0.00	1	31.83	0.00	1	22.16	0.00
	Riffle-pool	2	8.86	0.01	2	5.04	0.03	2	0.84	0.46	2	0.21	0.82

## 4. Discussion

### 4.1. Winter dynamics of functional diversity and redundancy of macroinvertebrates in the riffle stones and the pool sediments

Headwater streams usually show high spatial heterogeneity even in a few meters, creating mosaics (i.e., a sequence of riffles and pools) with different environmental conditions (Frissell et al., 1986; Mazão and da Conceição, 2016) and affecting aquatic macroinvertebrate assemblages (Vison and Hawkins, 1998; Heino et al., 2004; Clarke et al., 2008). As we expected, physical environmental characteristics and food resource availability were significantly different between riffles and pools in this study. Compared to riffles, pools were deeper, subjected to lower hydrological disturbance, containing more fine sediments and higher relative abundance of CPOM but less leaves. Under distinct environmental selection, both taxonomic and function diversities were higher in the stony substrates of riffles than the sedimental substrates of pools, but functional redundancy showed little difference, supporting partially our first hypothesis.

Physical disturbance and substrate type are key environmental factors affecting macroinvertebrate communities (Brown and Brussock, 1991; Roy et al., 2003). In our stream, water velocity and substrate composition significantly affected macroinvertebrate community composition in both riffles and pools. “None swim” and “Burrower” taxa, such as Chironomidae dominated the macroinvertebrates in pool sediments; while “weak swim” (e.g., *Taenionema*) dominated those in riffle stones. Food resources also constitute an environmental template for macroinvertebrates communities (Cummins and Klug, 1979; Beisel et al., 2000; Herbst et al., 2018). More abundant shredders mainly feeding on leave colonized in riffle stones than in pool sediments, because more leaves covered on riffle stones. More scrapers also colonized in riffle stones, because lots of rocks provide more surface habitats attached by diatom. And omre collector-filterers (e.g., *Hydropsyche*) were also been found in riffle stones, because they benefit from high water velocity that provides suspended organic matter. As a result, riffle stones supported a greater taxonomic and functional diversities of macroinvertebrates than pool sediments, which is similar with most comparative studies of stream macroinvertebrate communities between riffles and pools (Logan and Brooker, 1983; Brown and Brussock, 1991; Carter and Fend, 2001). Due to the synchronous change in both Simpson’s and Rao’s diversities of macroinvertebrates between riffle stones and pool sediments, there was no significant difference in functional redundancy between the two types of habitats.

It is common considered that functional trait composition has temporal stability in habitats with low environmental fluctuation and strongly changes only in the high environmental fluctuation (Statzner et al., 2004; Bêche et al., 2006). Freezing and thawing are two contrast environmental processes. Freezing decreases water temperature and hydrologic disturbance, whereas thawing increases water temperature and hydrologic disturbance. Significant variation of macroinvertebrates was found in functional trait composition between the two periods. During the pre-freezing period, “weak swim” group (e.g., *Taenionema*) became significantly abundant in riffle stones, indicating this group can escape from unfavorable habitats and adapt to considerable environmental changes caused by low temperature and freezing. Decrease in the relative abundance of “Erosional” group but increase in “Burrower” group indicated by their relative abundance in pool sediments suggest the adaptation of macroinvertebrates to low hydrological disturbance. During the thawing period, increase of “weak swim” group but decrease of “Burrower” group in pool sediments show their adaptation to high hydrological disturbance.

Taxonomic diversity, functional diversity and functional redundancy in riffle stones showed a low temporal variation, suggesting that freezing and thawing had less effects on riffle macroinvertebrates. In pool sediments, Simpson’s diversity increased significantly with the increased evenness during the thawing period, and functional redundancy increased significantly during the pre-freezing period, due to that Simpson’s diversity increased greater than Rao’s diversity. The seasonal change in biological diversity and functional redundancy of macroinvertebrate communities in pool sediments depend on its substrate stability. That is, the riverbeds in pool sediments that are mainly composed of sand and gravel are unstable and sensitive to hydrological disturbance (Duan et al., 2008). In comparison, riffle stones have a constitutional potential to maintain the temporal stability of structure and function of macroinvertebrate communities.

### 4.2. Litter patches altered functional diversity and redundancy of macroinvertebrate communities

Litterfall occurs mainly in streams within a short autumn period in boreal streams, they are intercepted in riffles or deposited in pools, forming litter patches (Egglishaw, 1964; Mackay and Kalff, 1969; Kobayashi and Kagaya, 2002). Being different from pure stony and sedimental substrates, litter patches over them largely modify the features of habitats, i.e., increasing habitat homogeneity by reducing weak hydrological disturbance but providing much more food resource (Cummins, 1974; Holomuzki and Hoyle, 1990; Dobson, 1994; Wallace et al., 1999). In our case, total litter abundance was found to influence significantly the community structure of macroinvertebrates.

In our riffles, the litter substrates attracted abundant shredders (e.g., *Gammarus*) by providing leaves for their feeding, as well as collector-gatherers (e.g., *Ephemerella* and Chironomidae) by accumulating much fine particulate organic matter, and abundant predators (e.g., *Utaperla*). Many studies have also found that litter retention largely determines the abundance of macroinvertebrates (Short et al., 1980; Dobson and Hildrew, 1992; Dobson, 1994; Wallace et al., 1999). However, not all taxa in our investigated riffles were attracted markedly by litter patches. More “Erosional” taxa (e.g., *Glossosoma* and *Cyrnellus*) and “Collector-filterer” taxa (e.g., *Hydropsyche* and *Simulium*) colonized in riffle stones, because they require high water flow for living (e.g., breathing or feeding). In general, litter patches support lower macroinvertebrate richness but higher density than stones. Due to higher community evenness in litter patches, Simpson’s diversity did not show significant difference between riffle litter and stony substrates. On the other hand, reduced environmental selection and limited competition promote trait clustering between species, resulting in lower functional diversity (Grime, 2006; Helmus et al., 2010). As our second hypothesis predicted, riffle litters had lower functional diversity but higher functional redundancy than riffle stones did.

The area of litter patches is larger in pools than in riffles, usually covering most of pool sediments. Due to the similar physical environment conditions, pool litter and pool sediment communities had similar taxonomic richness, density and Simpson’s diversity. However, high food resource reduced interspecific competition that decreased functional diversity, finally resulting in decrease in functional redundancy that supports our second hypothesis.

Litter patches are not a type of stable habitat, their food value and habitat feature change with the litter decomposition, which could affect macroinvertebrate communities. The decomposition of litter usually lasts for several months, generally in three phases: (1) leaching and initial rapid loss, (2) microbial conditioning, and (3) macroinvertebrate consumption and physical breakdown (Webster and Benfield, 1986; Gessner et al., 1999). Conditioning of microbes not only accelerates the decomposition of leaf litters but also changes the palatability of litters for shredders (Cummins et al., 1989; Gessner et al., 1999). With increase in litter palatability, litters could attract more abundance of macroinvertebrate (Cummins et al., 1989; Graça et al., 2001). According with this general temporal pattern, the macroinvertebrate density in this study indeed increased from the autumn to the freezing period in riffle litters and pool litters, despite the low temperature during the freezing period. This also suggests that macroinvertebrates could be more sensitive to food resources than to temperature changes. Many boreal stream studies also demonstrated that macroinvertebrates activity still active under low temperatures and may play a larger role in litter decomposition (Irons et al., 1994; Muto et al., 2011). After that, the macroinvertebrate density decreased during thawing period, due to decrease in litter nutrient content and increase in hydrological disturbance.

Under strong interspecific competition, the abundance of competitive taxa increases and its vulnerability decreases (see Ricotta et al., 2016). In our stream, *Taenionema* and *Rhyacophila* are both scrapers and overlap in their food niche. With highly competitive ability, *Taenionema* abundance increased a lot from the autumn to the freezing periods, and its vulnerability value significantly decreased from 0.21 to 0.14. In contrast, with a low competitive ability, *Rhyacophila* increased its vulnerability from 0.21 to 0.29. On the other hand, reduction in hydrological disturbance decreased environmental stress, which increased trait clustering between species in litter patches. As a result, functional trait composition and functional diversity of litter patch macroinvertebrates showed only slight change from the autumn to the freezing periods. During the thawing period, the reduced food tends to promote interspecific competition, but counteracted by increased hydrological disturbance, resulting in stable functional composition. As Simpson’s diversity and functional diversity did not change significantly during the studied four periods, the functional redundancy had temporal stability in the litter substrates of both riffles and pools, inconsistent with our third hypothesis.

In summary, compared with riffle stones and pool sediments, litter patches support not only higher total macroinvertebrate abundance but also higher functional redundancy which support an over-winter stability of functional trait composition and functional diversity.

## Data availability statement

The original contributions presented in this study are included in the article/Supplementary material, further inquiries can be directed to the corresponding authors.

## Author contributions

LW: conceptualization, methodology, investigation, writing—original draft, and writing—review and editing. LX and JL: methodology. LLW: methodology and editing. HY: conceptualization and funding acquisition. All authors contributed to the article and approved the submitted version.
