# Supplementary material for: Winter dynamics of functional diversity and redundancy of riffle and pool macroinvertebrates after defoliation in a temperate forest stream
Source: Front Microbiol. 2023 Mar 6;14:1105323. doi: 10.3389/fmicb.2023.1105323 (PMC10025292; doi:10.3389/fmicb.2023.1105323)
Supplement: Supplementary file 1 [file Data_Sheet_1.docx]

Supplementary Material

Winter dynamics of functional diversity and redundancy of riffle and pool macroinvertebrates after defoliation in a temperate forest stream

Lu Wang ^1,2^, Lixian Xia^1^, Jiaxu Li^2^, Linglin Wan^2*^, Haijun Yang^1*^

*** Correspondence:** Haijun Yang: yanghaijun@ynu.edu.cn

# Supplementary Tables

Table S1 Six functional traits of macroinvertebrates and their categories (codes).

| Trait | Trait category (code) |
| --- | --- |
| Voltinism | Semivoltine or Univoltine (Volt1); Bi- or multivoltine (Volt2) |
| Swimming ability | None (Swim1); Weak (Swim2); Strong (Swim3) |
| Shape | Streamlined (Shpe1); Not streamlined (Shpe2) |
| Rheophily | Depositional only (Rheo1); Depositional and erosional (Rheo2); Erosional (Rheo3) |
| Habitat | Burrower (Habi1); Climber (Habi2); Sprawler (Habi3); Clinger (Habi4); Swimmer (Habi5) |
| Trophic habit | Collector-gatherer (Trop1); Collector-filterer (Trop2); Herbivore (Trop3); Predator (Trop4); Shredder (Trop5) |

Table S2 Detritus area, detritus height, water depth and velocity in each period. Bold indicates significant difference (*p* < 0.05) between the pool litter patches and riffle litter patches.

|  | Autumn period | |  | | Pre-freezing period | |  | | Freezing period | |  | | Thawing period | |
| --- | --- | --- | --- | --- | --- | --- | --- | --- | --- | --- | --- | --- | --- | --- |
|  | pool litter patch | riffle litter patch | | pool litter patch | | riffle litter patch | | pool litter patch | | riffle litter patch | | pool litter patch | | riffle litter patch |
| Detritus area (cm^2^) | **3803.65 ± 789.01** | **463.30 ± 74.93** | | **4100 ± 1092.33** | | **339.40 ± 79.32** | | **3530.30 ± 1065.33** | | **429.00 ± 66.07** | | 2626.67 ± 1243.78 | | 480.06 ± 140.42 |
| Detritus height (cm) | **5.10 ± 0.78** | **11.60 ± 1.08** | | 7.10 ± 0.94 | | 7.80 ± 1.02 | | 5.60 ± 0.48 | | 4.00 ± 0.26 | | **9.00 ± 1.00** | | **18.56 ± 2.94** |
| Water depth (cm) | **16.45 ± 2.58** | **8.35 ± 0.63** | | 13.80 ± 2.17 | | 9.10 ± 1.08 | | **14.20 ± 1.72** | | **6.30 ± 1.02** | | **6.33 ± 1.33** | | **4.61 ± 0.42** |
| Velocity (m/s) | **0.04 ± 0.00** | **0.27 ± 0.05** | | **0.02 ± 0.00** | | **0.23 ± 0.04** | | **0.02 ± 0.02** | | **0.22 ± 0.05** | | **0.07 ± 0.03** | | **0.32 ± 0.04** |

Table S3 Mean density of each genus in riffle stones, pool sediments, riffle litters and pool litters in the four periods. RS–riffle stones, PS–pool sediments, RL–riffle litters, PL–pool litters.

|  | Autumn period | | | | Pre-freezing period | | | | Freezing period | | | | Thawing period | | | |
| --- | --- | --- | --- | --- | --- | --- | --- | --- | --- | --- | --- | --- | --- | --- | --- | --- |
|  | RS | PS | RL | PL | RS | PS | RL | PL | RS | PS | RL | PL | RS | PS | RL | PL |
| *Ephemerella* | 18.52 | 3.70 | 683.57 | 6.25 | 22.22 | 66.67 | 170.92 | 18.75 | 79.63 | 148.15 | 800.08 | 50.00 | 325.93 | 48.15 | 21.95 | 41.67 |
| *Siphlonurus* | 14.81 | 1.85 | 0.00 | 18.75 | 20.37 | 9.26 | 0.00 | 100.00 | 53.70 | 1.85 | 0.00 | 41.67 | 35.19 | 7.41 | 13.33 | 0.00 |
| *Ephemera* | 3.70 | 1.85 | 0.00 | 18.75 | 7.41 | 0.00 | 0.00 | 12.50 | 20.37 | 33.33 | 0.00 | 16.67 | 5.56 | 18.52 | 0.00 | 0.00 |
| *Utaperlar* | 37.04 | 33.33 | 424.75 | 50.00 | 53.70 | 20.37 | 146.03 | 218.75 | 90.74 | 7.41 | 912.58 | 158.33 | 50.00 | 12.96 | 20.28 | 33.33 |
| *Stavsolus* | 24.07 | 1.85 | 25.53 | 0.00 | 1.85 | 0.00 | 10.08 | 0.00 | 3.70 | 0.00 | 7.44 | 0.00 | 0.00 | 0.00 | 0.00 | 8.33 |
| *Megarcys* | 12.96 | 0.00 | 9.42 | 0.00 | 0.00 | 0.00 | 0.00 | 0.00 | 0.00 | 0.00 | 0.00 | 0.00 | 11.11 | 5.56 | 0.00 | 0.00 |
| *Amphinemura* | 0.00 | 0.00 | 11.16 | 6.25 | 0.00 | 0.00 | 0.00 | 6.25 | 37.04 | 12.96 | 0.00 | 0.00 | 188.89 | 44.44 | 0.00 | 0.00 |
| *Nemoura* | 0.00 | 0.00 | 0.00 | 0.00 | 0.00 | 0.00 | 0.00 | 0.00 | 1.85 | 0.00 | 31.25 | 0.00 | 51.85 | 14.81 | 94.75 | 25.00 |
| *Taenionema* | 1.85 | 0.00 | 217.61 | 0.00 | 437.04 | 22.22 | 303.08 | 12.50 | 1000.00 | 70.37 | 1848.39 | 41.67 | 31.48 | 7.41 | 113.33 | 216.67 |
| *Eucapnopsis* | 0.00 | 0.00 | 0.00 | 0.00 | 0.00 | 0.00 | 20.16 | 0.00 | 0.00 | 0.00 | 0.00 | 8.33 | 0.00 | 0.00 | 0.00 | 0.00 |
| Smicridae sp.1 | 9.26 | 0.00 | 0.00 | 0.00 | 0.00 | 0.00 | 0.00 | 0.00 | 0.00 | 0.00 | 0.00 | 0.00 | 0.00 | 0.00 | 0.00 | 0.00 |
| *Hydropsyche* | 118.52 | 22.22 | 16.60 | 0.00 | 37.04 | 7.41 | 17.86 | 0.00 | 175.93 | 66.67 | 0.00 | 0.00 | 70.37 | 0.00 | 15.28 | 8.33 |
| *Arctopsyche* | 31.48 | 7.41 | 63.89 | 6.25 | 24.07 | 7.41 | 6.49 | 6.25 | 94.44 | 3.70 | 0.00 | 8.33 | 14.81 | 0.00 | 0.00 | 0.00 |
| *Leptonema* | 0.00 | 0.00 | 0.00 | 0.00 | 0.00 | 0.00 | 0.00 | 0.00 | 1.85 | 0.00 | 0.00 | 0.00 | 0.00 | 0.00 | 0.00 | 0.00 |
| *Nemotaulius* | 0.00 | 0.00 | 0.00 | 18.75 | 0.00 | 0.00 | 0.00 | 0.00 | 3.70 | 0.00 | 0.00 | 0.00 | 0.00 | 0.00 | 0.00 | 0.00 |
| *Sphagnophylax* | 0.00 | 0.00 | 0.00 | 6.25 | 0.00 | 0.00 | 0.00 | 0.00 | 1.85 | 0.00 | 0.00 | 0.00 | 0.00 | 0.00 | 0.00 | 0.00 |
| *Pycnopsyche* | 0.00 | 0.00 | 0.00 | 31.25 | 0.00 | 0.00 | 0.00 | 0.00 | 0.00 | 0.00 | 0.00 | 0.00 | 0.00 | 0.00 | 0.00 | 0.00 |
| *Clostoeca* | 0.00 | 0.00 | 7.81 | 25.00 | 9.26 | 0.00 | 0.00 | 18.75 | 14.81 | 0.00 | 0.00 | 0.00 | 22.22 | 7.41 | 0.00 | 0.00 |
| *Hydatophylax* | 0.00 | 0.00 | 43.38 | 56.25 | 0.00 | 0.00 | 20.16 | 25.00 | 0.00 | 0.00 | 45.78 | 41.67 | 0.00 | 0.00 | 0.00 | 0.00 |
| *Oligostomis* | 0.00 | 0.00 | 0.00 | 6.25 | 0.00 | 0.00 | 0.00 | 0.00 | 0.00 | 0.00 | 0.00 | 0.00 | 0.00 | 0.00 | 0.00 | 0.00 |
| *Cyrnellus* | 33.33 | 9.26 | 7.18 | 0.00 | 83.33 | 0.00 | 0.00 | 0.00 | 35.19 | 0.00 | 0.00 | 0.00 | 1.85 | 0.00 | 0.00 | 0.00 |
| *Cyrnellus* | 37.04 | 5.56 | 0.00 | 0.00 | 33.33 | 3.70 | 0.00 | 0.00 | 24.07 | 7.41 | 0.00 | 0.00 | 48.15 | 7.41 | 0.00 | 0.00 |
| *Polyplectropus* | 0.00 | 0.00 | 31.60 | 37.50 | 3.70 | 0.00 | 17.86 | 6.25 | 0.00 | 0.00 | 53.60 | 16.67 | 0.00 | 0.00 | 6.67 | 0.00 |
| *Rhyacophila* | 5.56 | 0.00 | 7.18 | 0.00 | 0.00 | 0.00 | 6.49 | 0.00 | 1.85 | 0.00 | 7.44 | 0.00 | 0.00 | 0.00 | 0.00 | 0.00 |
| *Hyporhyacophila* | 0.00 | 0.00 | 7.18 | 0.00 | 0.00 | 0.00 | 0.00 | 0.00 | 0.00 | 0.00 | 0.00 | 0.00 | 0.00 | 0.00 | 0.00 | 0.00 |
| *Ecnomus* | 0.00 | 0.00 | 30.97 | 0.00 | 0.00 | 0.00 | 0.00 | 0.00 | 0.00 | 0.00 | 0.00 | 0.00 | 0.00 | 0.00 | 0.00 | 0.00 |
| Brachycentridae sp.1 | 0.00 | 0.00 | 7.81 | 0.00 | 0.00 | 0.00 | 0.00 | 0.00 | 0.00 | 0.00 | 0.00 | 0.00 | 0.00 | 0.00 | 0.00 | 0.00 |
| *Atopsyche* | 0.00 | 0.00 | 0.00 | 0.00 | 0.00 | 0.00 | 0.00 | 0.00 | 0.00 | 0.00 | 7.44 | 0.00 | 0.00 | 0.00 | 0.00 | 0.00 |
| Tanypodinae | 0.00 | 0.00 | 49.45 | 18.75 | 11.11 | 22.22 | 614.49 | 112.50 | 12.96 | 20.37 | 2064.15 | 83.33 | 0.00 | 0.00 | 288.08 | 83.33 |
| Orthocladiinae | 3.70 | 12.96 | 244.61 | 150.00 | 85.19 | 279.63 | 5009.08 | 387.50 | 516.67 | 659.26 | 3790.63 | 566.67 | 22.22 | 14.81 | 291.15 | 133.33 |
| Chironominae | 77.78 | 3.70 | 100.23 | 37.50 | 22.22 | 124.07 | 156.28 | 1006.25 | 177.78 | 116.67 | 282.74 | 566.67 | 64.81 | 44.44 | 37.23 | 100.00 |
| *Hexatoma* | 7.41 | 7.41 | 0.00 | 12.50 | 3.70 | 3.70 | 0.00 | 18.75 | 14.81 | 24.07 | 0.00 | 0.00 | 11.11 | 24.07 | 0.00 | 0.00 |
| *Antocha* | 7.41 | 7.41 | 0.00 | 0.00 | 42.59 | 5.56 | 0.00 | 0.00 | 27.78 | 3.70 | 0.00 | 0.00 | 22.22 | 1.85 | 8.61 | 0.00 |
| *Tipula* | 0.00 | 0.00 | 0.00 | 0.00 | 0.00 | 0.00 | 0.00 | 0.00 | 0.00 | 3.70 | 0.00 | 0.00 | 0.00 | 0.00 | 0.00 | 0.00 |
| *Pedicia* | 12.96 | 11.11 | 0.00 | 6.25 | 0.00 | 0.00 | 0.00 | 0.00 | 5.56 | 16.67 | 0.00 | 0.00 | 0.00 | 0.00 | 0.00 | 0.00 |
| *Dicranota* | 3.70 | 3.70 | 0.00 | 0.00 | 14.81 | 0.00 | 6.49 | 0.00 | 1.85 | 1.85 | 7.44 | 0.00 | 24.07 | 1.85 | 0.00 | 16.67 |
| *Telmatoscopus* | 0.00 | 0.00 | 0.00 | 6.25 | 0.00 | 0.00 | 0.00 | 0.00 | 0.00 | 0.00 | 0.00 | 0.00 | 0.00 | 0.00 | 15.28 | 0.00 |
| *Simulium* | 0.00 | 0.00 | 0.00 | 0.00 | 12.96 | 0.00 | 0.00 | 0.00 | 24.07 | 5.56 | 29.07 | 0.00 | 0.00 | 0.00 | 0.00 | 0.00 |
| *Culicoides* | 1.85 | 1.85 | 0.00 | 0.00 | 0.00 | 0.00 | 0.00 | 0.00 | 0.00 | 0.00 | 0.00 | 0.00 | 0.00 | 0.00 | 0.00 | 0.00 |
| *Limnophora* | 0.00 | 0.00 | 0.00 | 0.00 | 0.00 | 0.00 | 0.00 | 0.00 | 0.00 | 0.00 | 0.00 | 0.00 | 0.00 | 0.00 | 0.00 | 8.33 |
| *Osmylus* | 0.00 | 0.00 | 8.93 | 6.25 | 0.00 | 0.00 | 0.00 | 0.00 | 0.00 | 0.00 | 0.00 | 0.00 | 0.00 | 0.00 | 0.00 | 0.00 |
| *Pseudamophilus* | 161.11 | 144.44 | 137.95 | 18.75 | 50.00 | 100.00 | 29.56 | 18.75 | 66.67 | 55.56 | 99.38 | 16.67 | 20.37 | 5.56 | 6.67 | 0.00 |
| *Hydroporus* | 11.11 | 1.85 | 152.18 | 18.75 | 7.41 | 0.00 | 42.89 | 0.00 | 14.81 | 3.70 | 204.69 | 8.33 | 1.85 | 0.00 | 15.28 | 16.67 |
| *Pelonomus* | 0.00 | 0.00 | 0.00 | 0.00 | 0.00 | 0.00 | 5.68 | 0.00 | 0.00 | 0.00 | 0.00 | 0.00 | 0.00 | 0.00 | 0.00 | 0.00 |
| *Leptogomphus* | 0.00 | 0.00 | 7.81 | 12.50 | 0.00 | 0.00 | 0.00 | 0.00 | 0.00 | 0.00 | 0.00 | 8.33 | 0.00 | 0.00 | 0.00 | 0.00 |
| *Gammarus* | 137.04 | 24.07 | 751.46 | 68.75 | 122.22 | 29.63 | 320.73 | 31.25 | 200.00 | 18.52 | 340.19 | 16.67 | 131.48 | 20.37 | 21.95 | 25.00 |
| *Limnodrilus* | 7.41 | 0.00 | 0.00 | 0.00 | 0.00 | 0.00 | 0.00 | 18.75 | 0.00 | 0.00 | 0.00 | 0.00 | 0.00 | 0.00 | 0.00 | 8.33 |
| *Geora* | 3.70 | 0.00 | 0.00 | 0.00 | 5.56 | 0.00 | 0.00 | 0.00 | 0.00 | 0.00 | 0.00 | 0.00 | 0.00 | 0.00 | 0.00 | 0.00 |
| *Epeorus* | 11.11 | 1.85 | 0.00 | 0.00 | 3.70 | 0.00 | 0.00 | 0.00 | 35.19 | 3.70 | 0.00 | 0.00 | 62.96 | 5.56 | 0.00 | 0.00 |
| *Heptagenia* | 0.00 | 0.00 | 0.00 | 0.00 | 20.37 | 0.00 | 0.00 | 0.00 | 22.22 | 11.11 | 0.00 | 0.00 | 118.52 | 29.63 | 0.00 | 0.00 |

Table S4 Two-way ANOVA for period (Autumn period, Pre-freezing period, Freezing period and Thawing period) and habitat (Riffle stone and Pool sediment, Riffle stone and Riffle litter, Pool sediment and Pool litter) effects on Richness, Simpson, Rao and Functional redundancy (FR).

| ANOVA |  | df | F | P-value | Tukey multiple comparison |
| --- | --- | --- | --- | --- | --- |
| Riffle stone-Pool sediment | | |  |  |  |
| Richness | Period | 3 | 1.67 | 0.19 | N.A. |
|  | Habitat | 1 | 46.82 | 0.00 | Riffle stone > Pool sediment (each period) |
| Simpson | Period | 3 | 3.99 | 0.01 | Thawing period > Freezing period (pool sediment) |
|  | Habitat | 1 | 19.09 | 0.00 | Riffle stone > Pool sediment (autumn and freezing period) |
| Rao | Period | 3 | 3.27 | 0.03 | Thawing period > Freezing period (pool sediment) |
|  | Habitat | 1 | 33.59 | 0.00 | Riffle stone > Pool sediment (autumn, pre-freezing, and freezing period) |
| FR | Period | 3 | 5.67 | 0.00 | Pre-freezing period > Autumn (pool sediment) |
|  | Habitat | 1 | 11.59 | 0.00 | Riffle stone > Pool sediment (pre-freezing period) |
| Riffle stone-Riffle litter | | |  |  |  |
| Richness | Period | 3 | 0.14 | 0.94 | N.A. |
|  | Habitat | 1 | 13.99 | 0.00 | Riffle stone > Riffle litter (pre-freezing, freezing and thawing period) |
| Simpson | Period | 3 | 1.83 | 0.10 | N.A. |
|  | Habitat | 1 | 9.33 | 0.00 | Riffle stone > Riffle litter (thawing period) |
| Rao | Period | 3 | 1.42 | 0.08 | N.A. |
|  | Habitat | 1 | 197.43 | 0.00 | Riffle stone > Riffle litter (each period) |
| FR | Period | 3 | 1.42 | 0.07 | N.A. |
|  | Habitat | 1 | 539.57 | 0.00 | Riffle litter > Riffle stone (each period) |
| Pool sediment-Pool litter | | |  |  |  |
| Richness | Period | 3 | 0.57 | 0.63 | N.A. |
|  | Habitat | 1 | 0.01 | 0.94 | N.A. |
| Simpson | Period | 3 | 1.42 | 0.25 | N.A. |
|  | Habitat | 1 | 1.22 | 0.73 | N.A. |
| Rao | Period | 3 | 1.39 | 0.26 | N.A. |
|  | Habitat | 1 | 11.75 | 0.00 | Pool sediment > Pool litter (autumn and thawing period) |
| FR | Period | 3 | 0.70 | 0.56 | N.A. |
|  | Habitat | 1 | 40.89 | 0.00 | Pool litter > Pool sediment (each period) |

# Supplementary Figure


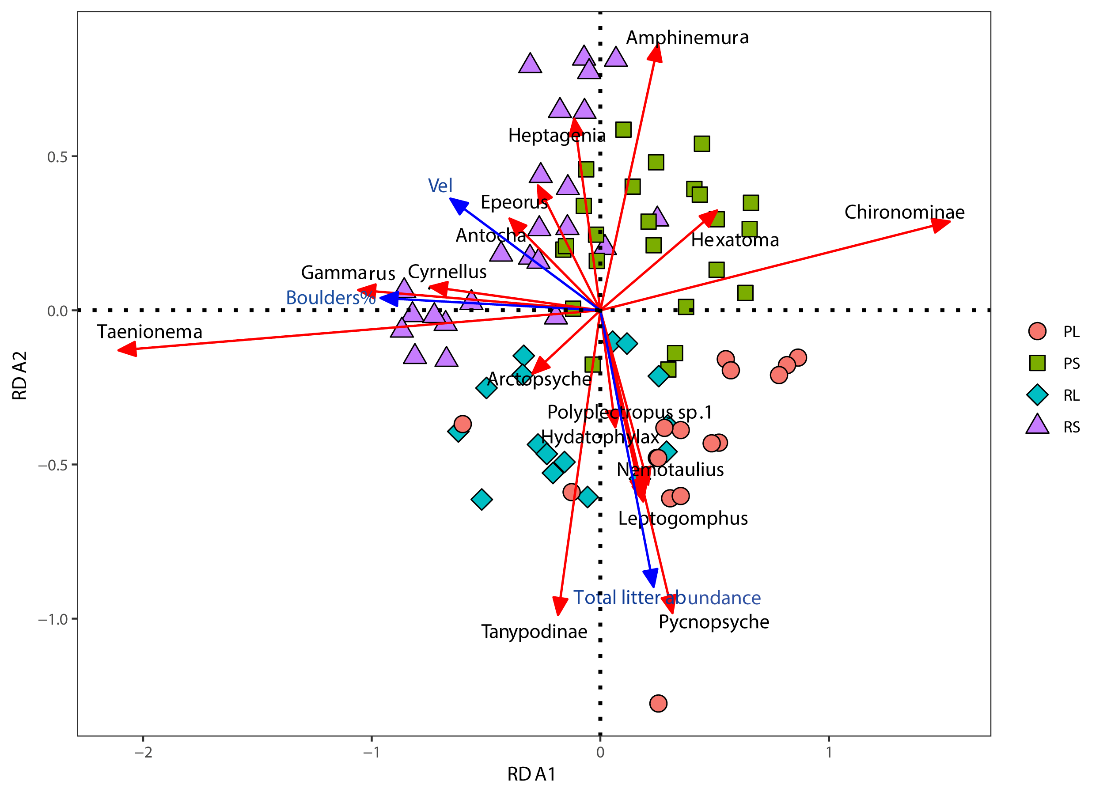


Figure S1 RDA plot of macroinvertebrates and environmental variables. The first and second axes were significant and respectively accounted for 6.81% and 4.63% of the variance in taxa-environment relationship (eigenvalue of first axis = 0.037, *F* = 5.881, *P* = 0.001; eigenvalue of second axis = 0.025, *F* = 3.999, *P* = 0.001). Only taxa with goodness-of-fit at least 0.1 were displayed. RS: Riffle stones; RL: Riffle litters; PS: Pool sediments; PL: Pool litters.
